# Case report: Dueling etiologies: Longitudinally extensive spinal cord lesion mimicking spinal cord infarct with simultaneous positive Lyme serology and amphiphysin antibody

**DOI:** 10.3389/fneur.2022.905283

**Published:** 2022-09-13

**Authors:** Marianna Kalaszi, Eoghan Donlon, Marzuki Wan Ahmad, Abdirahman Sheikh Mohamed, Peter Boers

**Affiliations:** Department of Neurology, University Hospital Limerick, Limerick, Ireland

**Keywords:** longitudinally extensive spinal cord lesion, transverse myelitis, spinal cord infarct, Lyme neuroborreliosis, amphiphysin antibody

## Abstract

**Background:**

Longitudinally extensive spinal cord lesions are challenging diagnostic entities as they are uncommon, but various etiologies can cause them.

**Case report:**

We report a case of a 55-year-old man with a past medical history of hypertension. He is an ex-smoker. He presented with chest pain, followed by right lower limb weakness, preceded by 2 weeks of constipation and voiding dysfunction. The examination revealed right lower limb mild flaccid paresis, absent reflexes, reduced anal tone, and urinary retention. His symptoms deteriorated over 24 h, and he developed severe flaccid paraparesis with impaired pinprick sensation below the T4 level. MRI spine showed an abnormal, non-enhancing signal in the anterior aspect of the spinal cord extending from the T4 level to the conus without associated edema. He was commenced on intravenous steroids and had significant improvement after one dose. The imaging was felt to be consistent with spinal cord infarction, and aspirin was started. The cerebrospinal fluid analysis showed elevated protein (0.8 mg/ml). Investigations for stroke and autoimmune pathologies were negative. The Lyme immunoblot confirmed intrathecal production of IgG to Borrelia antigens. The patient was started on ceftriaxone. The paraneoplastic screen identified amphiphysin antibodies. CT-TAP and PET-CT did not identify occult malignancy. The patient had a significant improvement over 2 months, strength was almost fully recovered, and autonomic functions returned to normal.

**Conclusion:**

We describe an unusual steroid-responsive, longitudinally extensive spinal cord lesion with radiological features of spinal cord infarct and a simultaneous finding of intrathecal Lyme antibodies and serum amphiphysin antibodies.

## Introduction

Myelopathy is defined as dysfunction of the spinal cord of any cause (1). It represents a heterogeneous group of disorders with distinct etiologies, clinical presentation, radiologic features, and prognoses (2). The clinical presentation and symptoms depend on the affected region of the spinal cord (3). The differential diagnosis is broad and includes metabolic, vascular, inflammatory, autoimmune, neoplastic, infective, traumatic, compressive, and idiopathic causes (4). Identifying the cause of myelopathy is critical, as delay in diagnosis and treatment could lead potentially to severe neurologic deficits (5).

## Case presentation

A 55-year-old right-handed man presented to the emergency department with sudden-onset central chest pain while walking lasting 20 min and resolved spontaneously, followed by gradually worsening weakness of the right lower limb. He was able to drive and walk 1 h after the onset of symptoms. He also reported altered bowel habit, constipation, and difficulty passing urine over the previous 2 weeks.

He had a past medical history of hypertension well-controlled on a beta-blocker. He worked as a delivery driver and was an ex-smoker with a 30-pack/year history. He had no recent travel, vaccination, or unusual contact with animals. However, he reported an insect bite 6 months ago associated with an erythematous bullseye-like rash.

On admission, he was afebrile and had stable vital signs. The electrocardiogram showed normal sinus rhythm with T-wave inversion in leads aVL and V1. Cardiovascular, respiratory, and abdominal examination was unremarkable. On neurological examination, he was alert and orientated. There was no cranial neuropathy. In the right lower limb, he had reduced tone and weakness, with hip and knee flexion 3/5, hip extension 3/5, knee extension 2/5, ankle dorsiflexion 3/5, and plantarflexion 2/5. Reflexes were absent in the right lower limb but otherwise normal elsewhere, and plantars were downgoing bilaterally. He had a normal sensory examination. There was no limb ataxia.

Over the next 24 h, he developed bilateral severe flaccid paresis of the lower limbs (right side: hip flexion 2/5, extension 3/5, abduction 3/5, adduction 3/5; knee flexion 2/5, extension 2/5, ankle dorsiflexion 1/5, and plantarflexion 2/5; left side: hip flexion 3/5, extension 4/5, abduction 3/5, adduction 3/5, knee flexion 3/5, extension 3/5, ankle dorsiflexion 4/5, and plantarflexion 4/5). He had bilaterally absent knee and ankle reflexes with upgoing plantar responses. The repeat sensory exam showed patchy impairment to pinprick sensation on the upper chest, and intact sensation of proprioception, light touch, and temperature. The bladder scan revealed urinary retention (410 ml post-void volume), and there was reduced anal tone with intact sensation.

Laboratory results on admission showed a normal complete blood count except for macrocytosis (106 fl). Liver and renal functions were in the normal range. C-reactive protein was normal (<5 mg/l), Erythrocyte sedimentation rate was 18 mm/h. Troponin serial data and D-dimer were negative. Blood alcohol level was high (161 mg/dl). Lipid profile showed elevated LDL-cholesterol (4.9 mmol/l) and low HDL-cholesterol (1.3 mmol/l). Hematinics (including serum vitamin B12, folic acid level, and iron studies), thyroid function, hemoglobin A1c, and serum angiotensin-converting enzyme level were in the normal range. SARS-CoV-2 RNA from nasopharyngeal swab and serum anti-SARS-CoV2 IgG were negative.

**Figure 1:**
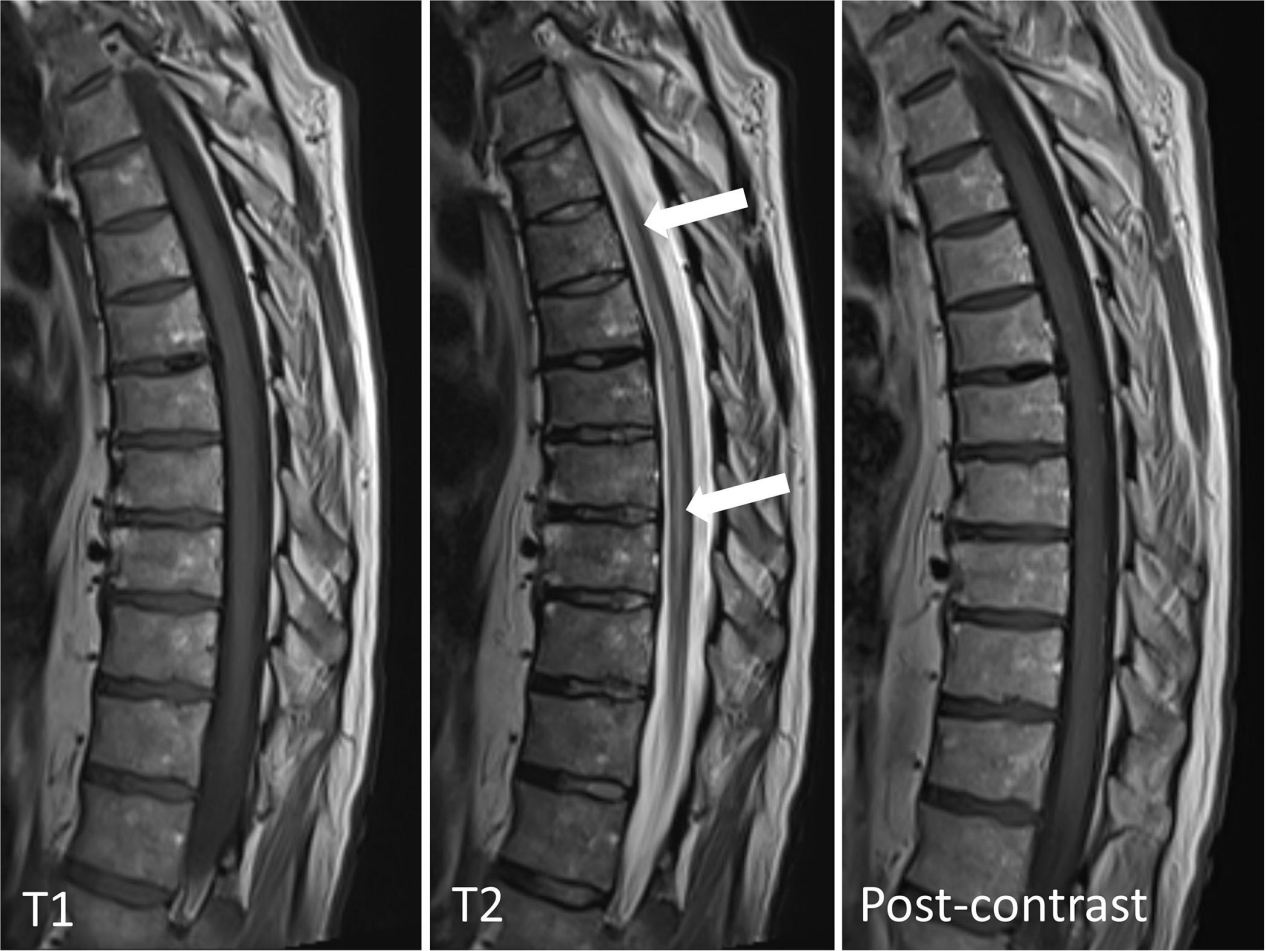
MRI of thoracic spine, sagittal view: non-enhancing high T2 signal in the anterior aspect of the spinal cord extending from T4 level to the conus without associated edema. The arrowheads point indicates the abnormal cord signal.

**Figure 2:**
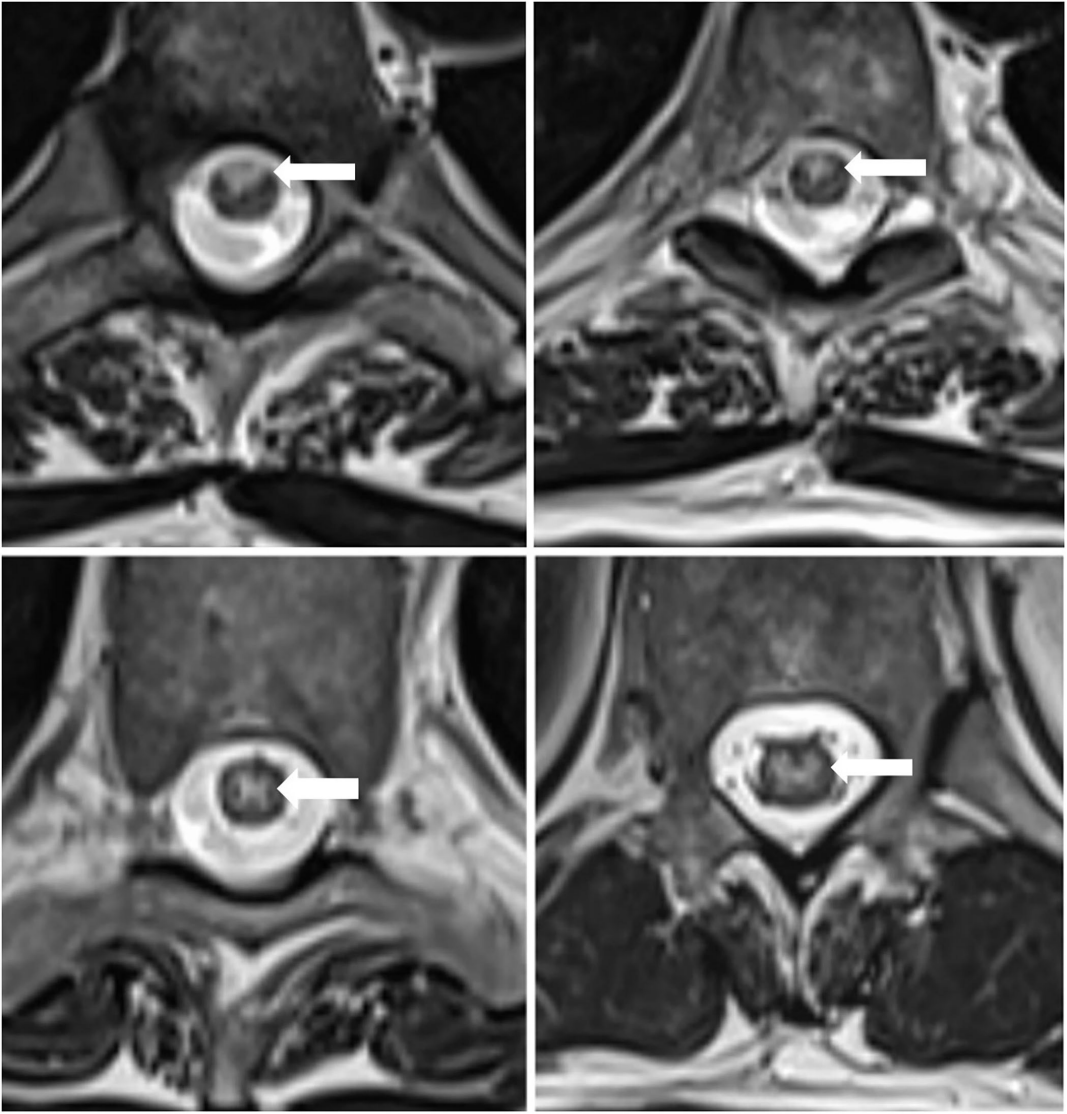
MRI of the thoracic spine, axial view: non-enhancing T2-hyperintensity in the anterior and centromedullar regions of the spinal cord. The arrowheads point indicates the abnormal cord signal.

Chest x-ray did not identify acute pathology. Computerized tomography (CT) of the brain showed mild cerebral atrophy and mild periventricular low density suggesting chronic microvascular disease. Magnetic resonance imaging (MRI) of the brain and the whole spine with contrast revealed an abnormal, increased T2 signal in the anterior aspect of the spinal cord beginning at the T4 level and extending to the conus without associated edema or contrast enhancement (Figures 1, 2). The additional diffusion-weighted imaging (DWI) of the spinal cord did not have a sufficient resolution.

The imaging findings were consistent with a longitudinally extensive transverse myelitis (LETM) of potential autoimmune, paraneoplastic, or infective etiology. Anterior spinal cord infarction was also considered within the differential diagnoses given the MRI characteristics (hyperintense T2 signal localized to the anterior and central portion of the cord with butterfly shaped appearance) and the acute symptom onset with associating chest pain.

On the 3rd day of his admission, he was started on daily 1 g intravenous (IV) methylprednisolone, and he had a noticeable improvement of his muscle strength after the first dose. He finished a 3-day course of IV methylprednisolone. He was also commenced on daily oral 300 mg aspirin on the 4th day of the admission.

The repeat MRI of the whole spine on day 10 of his admission showed interval resolution of the T2 signal hyperintensity in the lower cord and conus.

CT of the thorax, abdomen, and pelvis (CT-TAP) with contrast showed no abnormality; 24-h ECG, ambulatory blood pressure monitor (ABPM), and echocardiogram showed normal studies.

The cerebrospinal fluid (CSF) analysis showed normal leukocyte count (<5/ml), normal glucose (3.6 mmol/l, paired serum glucose 6.1 mmol/l), and elevated protein (0.8 g/L, normal range: 0.15–0.45 g/L). CSF oligoclonal band (OCB) with paired serum was negative. Polymerase chain reaction (PCR) assays of the CSF sample for bacterial (*Escherichia coli* K1, *Haemophilus influenzae, Listeria monocytogenes, Neisseria meningitis*, beta-hemolytic *Streptococcus* Group B, and *Streptococcus pneumoniae*), viral (cytomegalovirus, enterovirus, herpes simplex viruses 1 and 2, human herpesvirus 6, human parechovirus, and varicella zoster), and yeast (Cryptococcus) neoformans/gattii infections were negative.

Vasculitis and autoimmune tests (including antinuclear, ANA, and antineutrophil cytoplasmic, ANCA), cardiolipin, beta-2-glycoprotein, anti-myelin oligodendrocyte glycoprotein (MOG), aquaporin-4, anti- CASPR2, NMDA- receptor, and anti- Lgil antibodies) were negative. Serology for different infectious diseases (human immunodeficiency viruses 1 and 2, syphilis, cytomegalovirus, Epstein-Barr virus, varicella zoster) returned negative except for the enzyme-linked immunosorbent assay (ELISA) for Lyme C6 antibody, which showed a strong positive result (3.02, normal 0–0.9), but the confirmatory serology immunoblot result was negative. As there was a high clinical suspicion for possible Lyme neuroborreliosis, he was started on 2 g ceftriaxone intravenously twice daily on day 12 of his admission, and a CSF sample was sent for Lyme immunoblot testing. Later, this identified the presence of intrathecal immunoglobulin G (IgG) against two specific Borrelia antigens, p21 and VlsE. He finished a 21-day course of IV ceftriaxone. A paraneoplastic neuronal screen was also carried out and revealed the presence of the amphiphysin antibody. Other onconeural antibodies (anti-Hu, Yo, Ri, Ma2, CV2/CRMP, Zic-4, Sox-1, Tr, titin, and Recoverin) were negative. He underwent positron emission tomography CT (PET-CT), but it did not identify any occult neoplasm.

**Figure 3:**
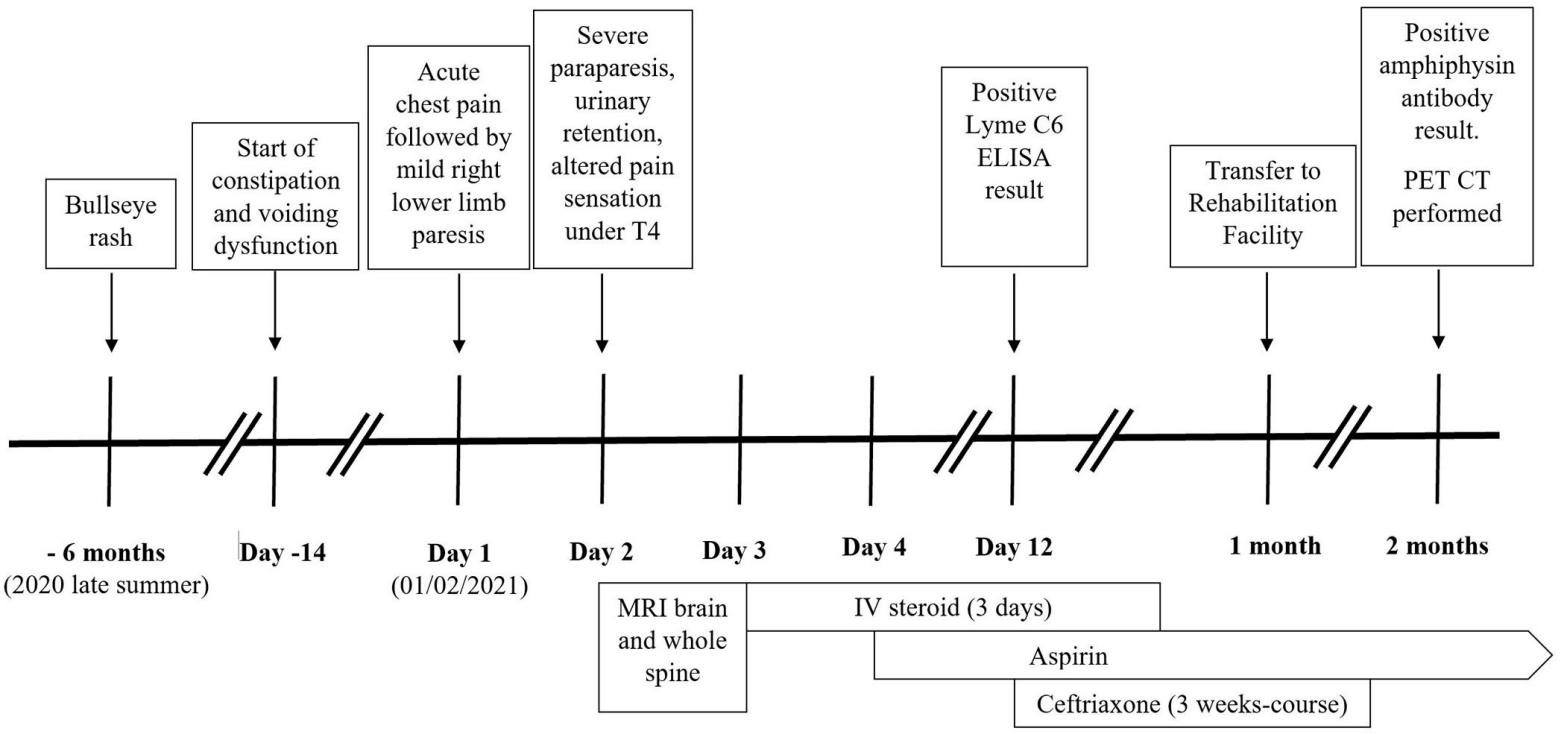
Case timeline.

The patient improved with rehabilitation and was discharged to a rehabilitation facility after 1 month of hospital admission. He was able to mobilize with a walking frame under supervision with minimal residual right lower limb weakness in 4 weeks. After 6 weeks, he was able to walk unaided with a stick, and bowel and bladder functions were fully recovered (Figure 3).

## Discussion

Longitudinally extensive spinal cord lesions (LETMs) are defined as extensive involvement of the spinal cord where an abnormal hyperintense T2-weighted signal affects at least three vertebral segments (6). The patient presented with symptoms, signs, and MRI scan features of an LETM. The clinical presentation was consistent with spinal cord infarction, autoimmune or idiopathic LETM. However, following extensive workup, several other possible causes were found, including infectious (Lyme neuroborreliosis), inflammatory and paraneoplastic etiologies.

### Spinal cord infarction (SCI)

Two recent large retrospective studies showed that patients who were initially diagnosed with idiopathic transverse myelitis often had alternative myelopathy diagnoses, and vascular cause was found to be the second most common after inflammatory etiology (7, 8). Various vascular mechanisms can lead to myelopathies including arterial ischemia, venous ischemia or congestion, hematomyelia, and extraparenchymal hemorrhage (9). Arterial ischemia can be divided into spinal cord transient ischemic attack, spontaneous spinal cord infarction, and periprocedural spinal cord infarction (9). SCI is a rare presentation due to the extensive collateral vascular supply of the spinal cord (10). The incidence is estimated at 3.1 per 100,000 people (9), representing 5–8% of acute myelopathies (11). Although SCI is more common in population with cardiovascular risk factors, it can also affect younger patients through various mechanisms (fibrocartilaginous embolism, coagulopathies, vertebral dissection, surfer's myelopathy, and cocaine use) (10). Rarely, systemic, infectious and paraneoplastic vasculitis can also affect the spinal cord vasculature leading to spinal cord infarction (12).

Symptoms of spinal cord infarction depend on the effected spinal cord region. Around 65% of spinal cord infarcts occur in the lower thoracic region (11). Insult of the anterior spinal artery territory can cause bilateral corticospinal tract deficit, lower motor neuron sign at the lesion level, loss of pain and temperature. Posterior spinal artery infarct results in dorsal column dysfunction (9).

Most commonly, patients have a sudden onset of symptoms (9). Around 70% of patients report acute back, chest, neck, or limb pain before the neurological deficit. This feature can be helpful to clinicians, because acute pain is atypical in myelitis (13). Patients usually develop flaccid muscle weakness with absent reflexes first. Upper motor neuron signs typically appear over time (11).

The typical MRI feature is T2 hyperintense signal in the territory of the effected artery. Most commonly, an anterior “pencil-like” lesion on sagittal sequences and an “owl/snake-eye” pattern on axial sequences are seen. These correspond to involvement of anterior horn cells, which are most vulnerable to ischemia (4). Significant spinal cord edema or contrast enhancement is unusual acutely (9). Diffusion-weighted imaging also recommended to be performed, but it has technical limitation, signal change can take days to evolve and its sensitivity is only 50% to 70% (9). Spinal cord infarction CSF analysis can show mild to moderate protein elevation (9).

This patient's MRI scan was suggestive of spinal cord infarction, and this was correlated with the initial clinical presentation. His CSF was also in keeping with this diagnosis. However, the clinical deficit seen in a spinal cord infarction would not typically improve with intravenous steroids, as what occurred in this patient (12).

### Lyme neuroborreliosis and Lyme-associated myelitis

Lyme disease is an infectious disorder caused by tick-borne spirochetes of the *Borrelia burgdorferi sensulato* complex (*B. burgdorferi sensustricto, B. garinii, B. afzelii, B. spielmanii, B. bavariensis*, and *Candidatus B. mayonii*) (14, 15). Involvement of the nervous system is referred to as Lyme neuroborreliosis (LNB) (14). More than 95% of neurological presentations are considered as early Lyme neuroborreliosis with symptoms presenting <6 months after the infection. A minority (5%) of the cases count for late LNB with a disease duration between 6 months and several years (16). Involvement of the central nervous system (CNS) in late LNB is rare and only occurs in 4% of cases, including myelitis, cerebral vasculitis, stroke-like signs due to occlusive vasculitis and cerebral infarction, chronic progressive encephalitis, encephalomyelitis with tetraspasticsyndrome, and spastic–ataxic gait disorder (16–18).

Most guidelines recommend a two-tiered testing protocol to confirm Lyme disease that relies on an initial sensitive screening test (commonly an ELISA) followed by a confirmatory immunoblot (IB) after a positive or equivocal ELISA result (17, 19).

Most frequently, LNB is associated with elevated CSF cell count (typically 10–1,000 leucocytes/ mm^3^) and elevated CSF protein. A normal CSF cell count is rare, but it can be present (16).

CSF serology testing is also recommended by most guidelines. The presence of Bb-specific antibodies (in comparison to serum values and normalized for the state of the blood–brain barrier, called *Borrelia*-specific CSF/serum antibody index) is the evidence of intrathecal antibody production and considered the traditional diagnostic gold standard (17). The ESGBOR (the ESCIMID Study Group for Lyme borreliosis) guideline specifies that the diagnostic sensitivity of the intrathecal antibody synthesis is about 80% in a shorter disease duration (<8 weeks) and nearly 100% in a longer disease duration (20).

MRI findings in Lyme myelopathies show high variability. Lindland et al. (21) found that the cervical spinal cord is affected most frequently, lumbosacral myelitis is less common, and the majority is longitudinally extensive and centrally or slightly anteriorly located. Enhancement patterns ranged from no enhancement to nodular or diffusely extensive contrast enhancement (21).

A literature review by Kaiser et al. summarized the findings of published case reports of transverse myelitis secondary to Lyme disease from 1989 to 2018 with a total number of 48 (3). In this cohort, high variability of spinal cord lesion localization and degree of involvement was noted. The spinal MRI demonstrated spinal cord edema in 12 cases, and 1 case had a poliomyelitis pattern with anterior horn involvement. Seven of the cases discussed in their review had similar thoracic and lumbar longitudinally extensive spinal lesions similar to our patient.

In addition to the literature review by Kaiser et al., we found seven additional reported cases of Lyme-associated transverse myelitis in the adult population published in English literature (22–28). Our literature search involved articles on PubMed written in the English language.

It is recognized that an initial positive result of ELISA testing for Lyme disease may represent a false positive result, and this needs to be confirmed by immunoblot testing. In this patient's case, the confirmatory serology test was negative. In addition, the CSF white cell count was < 5/mm^3^, and Lyme neuroborreliosis does not typically improve with steroid treatment (16). However, his CSF testing later identified intrathecal immunoglobulins directed against two *Borrelia*-specific antigens, which again raised the possibility of this being due to Lyme infection. A 21-day course of IV ceftriaxone was therefore started.

### Myelopathy associated with amphiphysin antibody

The amphiphysin antibody is considered a well-characterized paraneoplastic antibody commonly associated with small cell lung cancer and breast cancer (29). Stiff person syndrome is the most well-known clinical presentation with amphiphysin antibody, but cases of rapidly progressive myelopathy and LETMs have been also reported (30). A comprehensive review of amphiphysinrelated neurological presentations by Pittock et al. (30) looked at the results of 120,000 patients who were tested for paraneoplastic antibodies. The amphiphysin antibody was detected, in total, 71 of the patients. The neurological presentations included, neuropathy, encephalopathy, myelopathy, stiff-man phenomenon, and cerebellar syndrome. Myelopathy was confirmed in 17 patients. Out of the 71 patients, cancer was found in 50 patients, proven histologically in 46 patients, and most of them had small cell lung carcinoma or breast cancer. The neurological presentation preceded cancer detection in 90% of the patients. In the case of 20 patients, cancer was not identified in the initial investigation but was detected during the 2 years of surveillance. Another retrospective study carried out by Flanagan et al. looked at the results of 31 patients who presented with isolated myelopathies and either had a known coexisting cancer or tested positive for paraneoplastic antibody with strong cancer association (31). All the patients presented with ether subacute or insidious onset. Nine of the patients had amphiphysin antibodies. Spinal MRI showed T2 signal abnormalities in 20 patients (65%), and 14 patients had a longitudinally extensive abnormality involving more than 3 vertebral segments. In 15 patients, it showed a symmetric tract or gray matter-specific signal abnormality, and gadolinium enhancement was present in 13 patients. Cancer was confirmed in 87% of the patients. Myelopathy preceded cancer diagnosis in 18 patients, and seven patients were initially considered to have primary progressive multiple sclerosis.

This patient was assessed for an occult malignancy, including CT and PET scanning, which was negative. Patients with a paraneoplastic disorder usually only show some improvement with treatment of the underlying malignancy; however, a temporary improvement may occur with immune therapy, including steroid treatment. This improvement would not usually be sustained, in the absence of definitive treatment of the malignancy (30, 31). It is possible for paraneoplastic neurological disorders to present some time before the causative malignancy can be identified. Therefore, continued interval surveillance for malignancy is indicated in these cases, and that will be conducted in our patient, too.

## Conclusion

Acute myelopathies represent a heterogeneous group of disorders with distinct etiologies and clinical and radiologic features (2). We describe the diagnostic challenges of a steroid-responsive longitudinally extensive spinal cord lesion with radiological features of anterior spinal infarct and a simultaneous finding of intrathecal Lyme and serum amphiphysin antibodies. According to our knowledge, no similar case has been described in the literature where all the rare myelopathy etiologies were found in the same individual and could explain the clinical presentation. The MRI findings (anterior involvement and butterfly-shaped T2 hyperintense signal), acute paresis, associated chest pain, and significant cardiovascular risk factors supported a diagnosis of spinal cord infarction. In parallel, our patient also reported a bullseye rash 6 months before his acute symptoms, complained of autonomic dysfunction 2 weeks prior to the acute symptoms, and presence of intrathecal IgG against two specific *Borrelia* antigens (p21 and VlsE), which confirmed Lyme neuroborreliosis. He had an excellent clinical response to steroids and ceftriaxone with steady recovery during his admission. These features are supportive for LETM caused by late Lyme neuroborreliosis or possible Lyme-associated vasculitic infarction of the spinal cord. Finally, the amphiphysin antibody was also detected in his serum sample, which can be associated with isolated LETM, and the presence of the amphiphysin antibody can precede cancer detection up to 2 years (30). Although the CT TAP and PET- CT during his admission did not identify any occult neoplasm, he will need a close follow-up in the future. In this man's case, it is not possible to say with absolute certainty whether there was one unique cause of his presentation, with the other possible causes being co-incidental or false positive, or whether in fact he had the most unusual coincidence of three different causes of his LETM. The improvement with intravenous steroids argues against this being solely due to isolated spinal cord infarction; the previous rash and CSF immunoglobulin findings are suggestive of Lyme etiology, which was treated with appropriate antibiotic therapy, and the amphiphysin antibody result means that he will need an ongoing follow-up and surveillance for an occult malignancy.

## Data availability statement

The original contributions presented in the study are included in the article/supplementary material, further inquiries can be directed to the corresponding author.

## Ethics statement

Written informed consent was obtained from the individual for the publication of any potentially identifiable images or data included in this article.

## Author contributions

MK conceived of and drafted the manuscript. ED, MA, AM, and PB reviewed, revised, and edited the manuscript. All authors were involved in the care of the patient and read and approved the final version of the manuscript.

## Conflict of interest

The authors declare that the research was conducted in the absence of any commercial or financial relationships that could be construed as a potential conflict of interest.

## Publisher's note

All claims expressed in this article are solely those of the authors and do not necessarily represent those of their affiliated organizations, or those of the publisher, the editors and the reviewers. Any product that may be evaluated in this article, or claim that may be made by its manufacturer, is not guaranteed or endorsed by the publisher.
